# Adipose Tissue Grafting for the Treatment of Morphea En Coup De Sabre: A Simple Filler or an Emerging Cellular Therapy?

**DOI:** 10.7759/cureus.30358

**Published:** 2022-10-16

**Authors:** Mounia El Omari, Malak Debbarh, Mohamed Amine Lakhdari, Zineb Basri, Rita Ait Benhamou

**Affiliations:** 1 Plastic and Reconstructive Surgery, Cheikh Khalifa International University Hospital, Mohammed VI University of Health Sciences, Casablanca, MAR; 2 Medicine, Cheikh Khalifa International University Hospital, Mohammed VI University of Health Sciences, Casablanca, MAR

**Keywords:** adipocytes transplant, lipofilling, scleroderma, en coup de sabre, morphea, autologous fat grafting

## Abstract

Linear scleroderma en coup de sabre is a rare chronic autoimmune disease. This form of localized scleroderma manifests through an inflammatory phenomenon of collagen overproduction and extracellular matrix destruction, affecting the skin and subcutaneous tissues of the face. This active pathological process is challenging to control and often goes unnoticed at its early stage. The sequelae manifest as an alopecic frontal scar lesion, causing the patient significant aesthetic and psychological damage.

In this study, we report two clinical cases in which aesthetic sequelae were treated by autologous fat transplantation. We found encouraging results, with a global aesthetic improvement of 86.2% measured by a jury of medical specialists and outcome stability through a nine-year follow-up for one patient.

Autologous fat transfer is excellent for treating stabilized en coup de sabre morphea, not only for its filling abilities but also for its regeneration activating and inflammation regulating abilities, which could open new prospects for curative treatment of this pathology.

## Introduction

The term morphea is a Greek word that means shape or localized scleroderma in Anglo-Saxon literature. It covers various dermatological manifestations that have a common pathological hardening of some tissues. It is characterized by sclerosis, leading to fibrosis of the dermis and underlying tissues, without any known etiology [1].

Linear morphea en coup de sabre is a separate entity. It is exceedingly rare and predominant in the pediatric population. It manifests clinically as a typical facial deformity. Its extremely conspicuous appearance leads to aesthetic and psychological problems, which are more devastating in children and young women [2]. Several medical treatments to stabilize the evolution of this pathology have been described without any real consensus. However, to date, no curative treatment has been discovered [1].

We present two cases of a seven-year-old boy and a 25-year-old woman with stabilized morphea en coup de sabre treated with autologous fat graft, in which through the surgical handling of the aesthetic sequelae of this pathology, we tried to assess the relevance of an autologous adipocyte graft to restore the volumes and widen the horizons on the possibility of using cellular therapy.

## Case presentation

Patient 1

Patient 1 was a seven-year-old child with a history of allergic asthma but without any form of trauma or family history of autoimmune diseases. At the age of eight months, the parents noted the spontaneous appearance of a linear paramedian skin lesion that was less than 1 cm (about 0.39 in) wide, atrophic, dyschromic, and erythematous. The lesion progressively worsened with enlargement and downward extension toward the internal canthus and root of the nose; it extended upwards toward the scalp, accompanied by total alopecia opposite the lesion.

**Figure 1:**
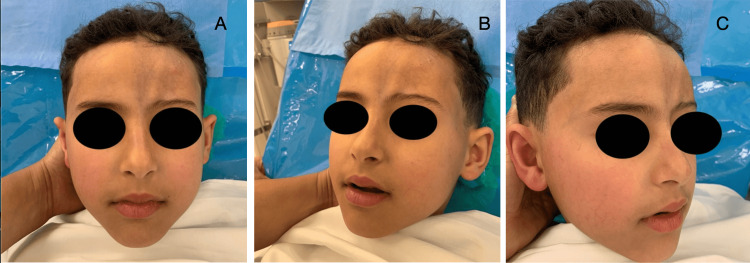
Preoperative front (A), 3/4 left (B), and right (C) photographs of patient 1

On examination, there was a left paramedian longitudinal frontoparietal alopecic patch, 8.2 cm (about 3.23 in) long x 4.8 cm (about 1.89 in) wide. On the face, there was a triangular patch with a left upper paramedian base extending from the forehead to the root of the nose, and sparing the internal canthus and the head of the eyebrow. At this level, the skin tissue was atrophic and hyperpigmented, forming a linear en coup de sabre depression that distorted the contour and profile of the face (Figure 1). A diagnosis of linear en coup de sabre was made in view of the typical clinical appearance.

The CT showed no bone involvement; nevertheless, a frontal skin biopsy was performed and the diagnosis was confirmed intraoperatively.

Patient 2

Patient 2 was a 25-year-old woman with a history of allergic rhinitis and asthma, but with no family history of a similar condition or of any autoimmune disease. She reported a closed trauma to her forehead without apparent lesions at the age of 18. A year later, she noticed a band-like thinning of hair on the right forehead, eventually leading to alopecia. Six months later, she noted the appearance of hyperpigmentation, and a right paramedian depression on the forehead, which progressively widened and extended down toward the root of the nose. The evolution was marked by spontaneous stabilization of the process for three years. The apparent aesthetic damage caused by this lesion and its psychological and social impact motivated her to consult a physician.

**Figure 2:**
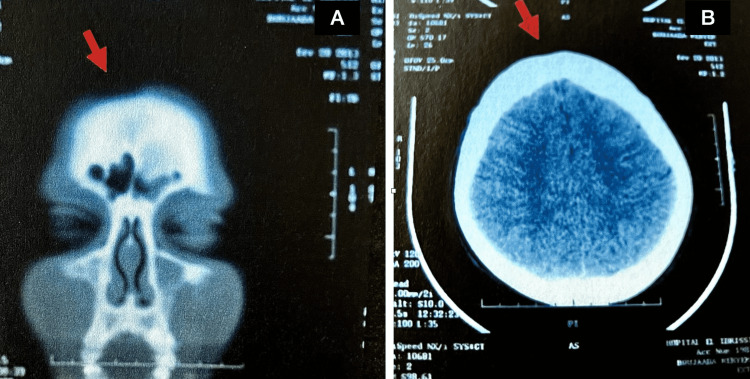
CT scan of patient 2 with no bone invasion (A) Coronal section and (B) axial section. Arrow: discrete frontal depression.

**Figure 3:**
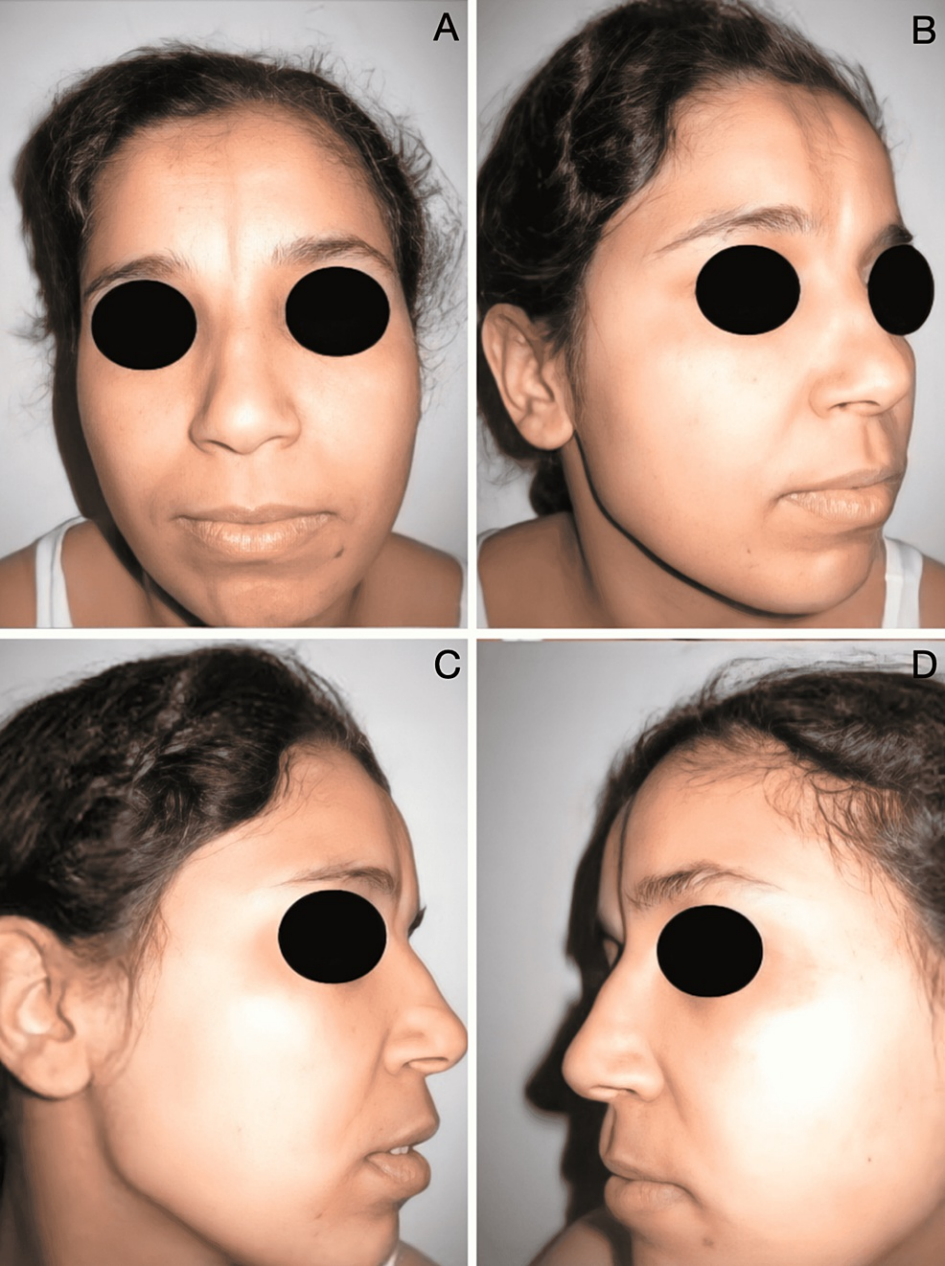
Preoperative front (A), 3/4 left (B), profile left (C), and profile right (D) photographs of patient 2

On examination, she had a right longitudinal, paramedian, frontoparietal, and alopecic plaque of 4 cm (about 1.57 in) x 6.5 cm (about 2.56 in). At the level of the face, there was a triangular plaque of 3.5 cm (about 1.38 in) x 9 cm (about 3.54 in), with a right paramedian superior base, extending from the forehead to the root of the nose, sparing the internal canthus and the head of the eyebrow. At this level, the cutaneous tissue was atrophic and hyperpigmented, forming a linear en coup de sabre depression distorting the shape and profile of the face. The diagnosis of linear en coup de sabre was made because of its typical clinical appearance (Figures 2, 3).

**Figure 4:**
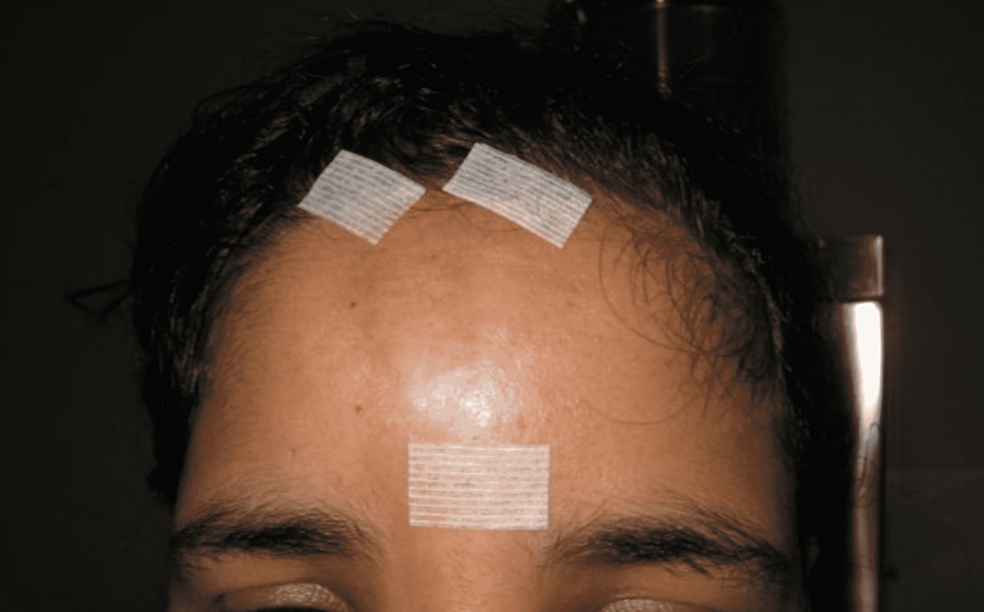
Early postoperative aspect of lipofilling in patient 2

A pathological examination performed postoperatively on the excision specimen confirmed the diagnosis (Figure 4).

Methods

Methods of Intervention

After informing the patients about the chosen treatment, they consented to its duration, benefits, constraints, and possible complications. For both patients, surgical treatment was indicated at the stage of sequelae, after the stabilization of the lesions previously treated by an internist. Perioperative and postoperative antibiotic prophylaxis with ciprofloxacin was prescribed. The intervention was performed following the technique described by Dr. Sydney Coleman [3].

**Figure 5:**
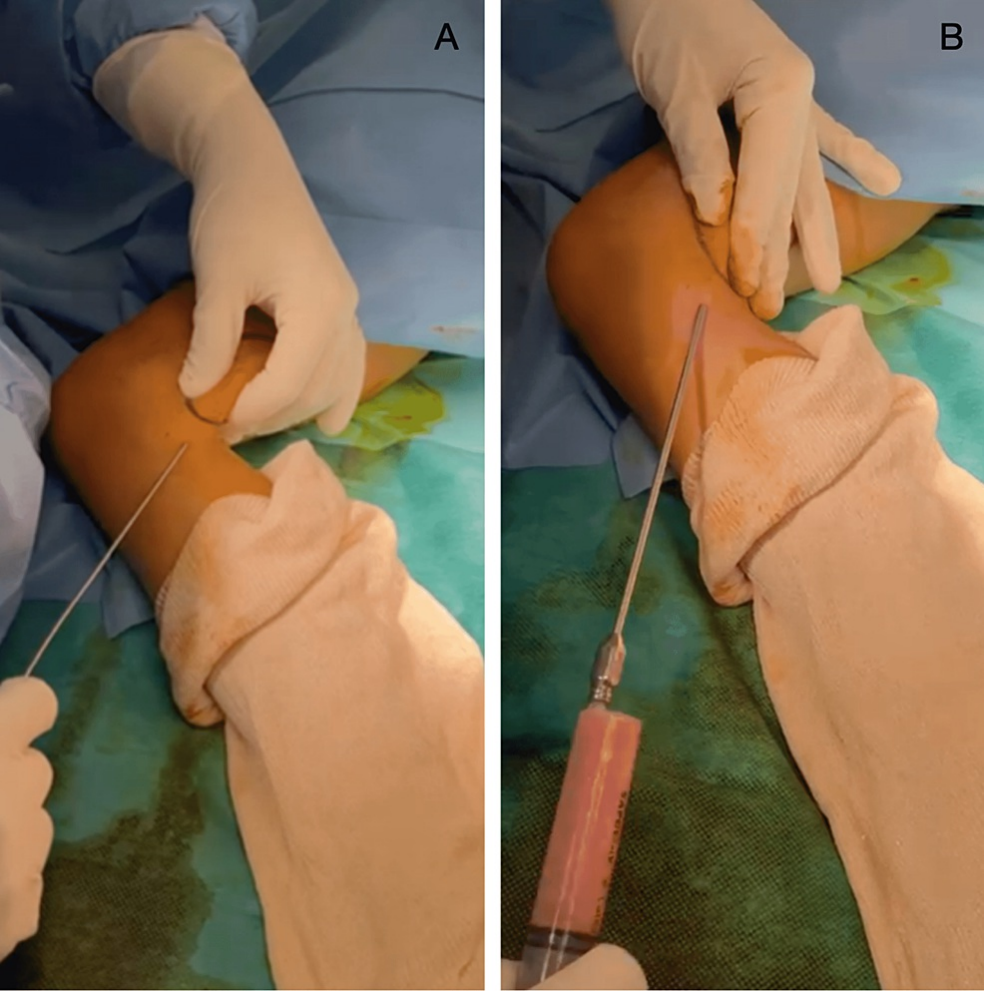
Fat collection (A and B) from the medial part of the knee of patient 1 (a constant donor site even in the pediatric population)

**Figure 6:**
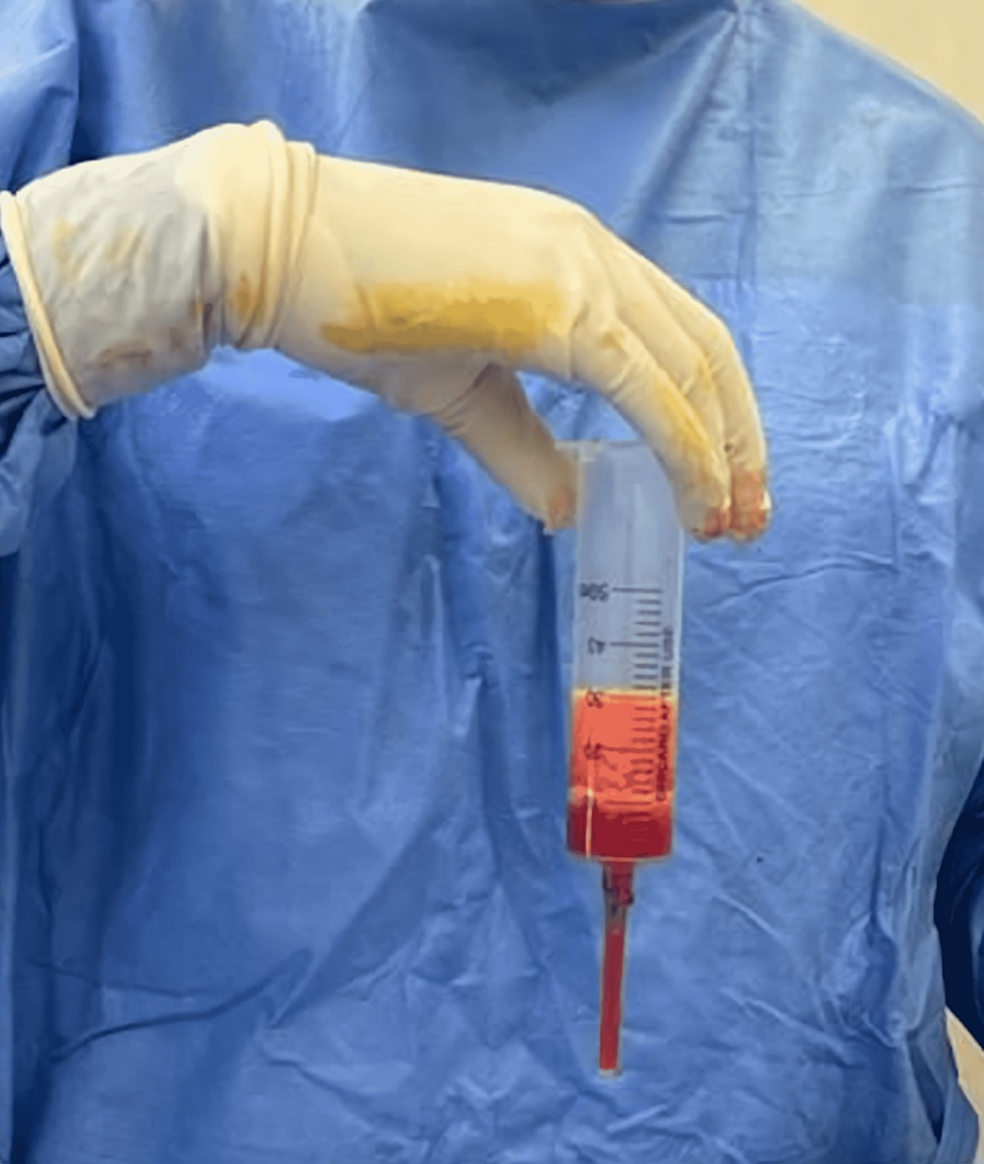
Triple layer appearance of sampling

**Figure 7:**
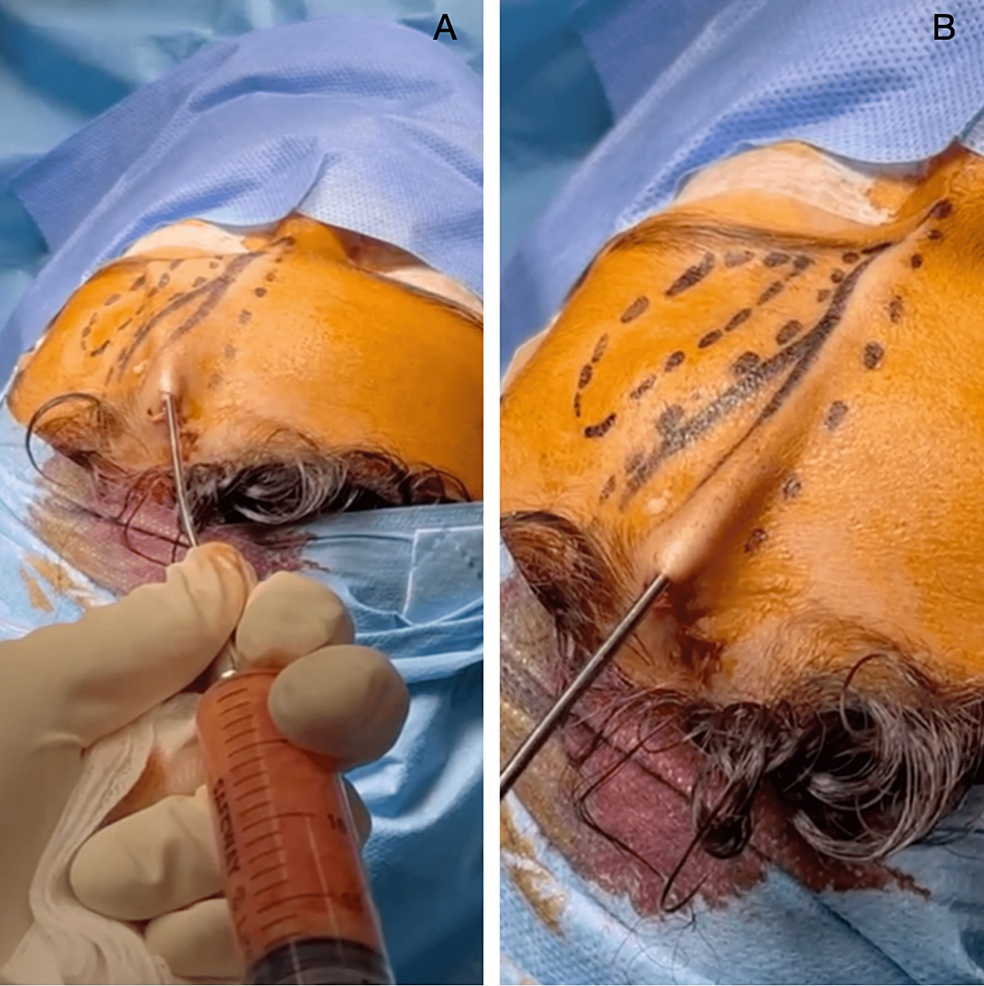
Reinjection at the recipient site of patient 1

For our first patient, 15 cc of pure fat was collected by gentle liposuction from the medial part of the knee and reinjected at the frontal and glabellar levels in one session, while the second patient received surgical treatment of alopecia: excision and reconstruction by pre-expanded hair flap, in addition to the autologous fat injection of 25 cc collected inferior to the umbilical region by gentle liposuction and reinjected to the recipient site all in one session (Figures 5-7).

Methods of Observation

Data were collected from the medical record and through postoperative follow-up in the third month and every six months thereafter. Photographs were taken before and after the procedure.

Evaluation Methods

A questionnaire was sent to both patients for self-evaluation. The questions concerned the sequelae in both the sampled area and the healing results obtained in the treated area.

A specialist jury composed of dermatologists, plastic surgeons, and internists objectively assessed the results obtained based on our patients’ pre- and postoperative photographs.

We chose the Localized Scleroderma Cutaneous Assessment Tool (Lo-SCAT) scale as a scoring tool to closely monitor the pathological activity and evolution of the lesions in our patients.

Results

**Figure 8:**
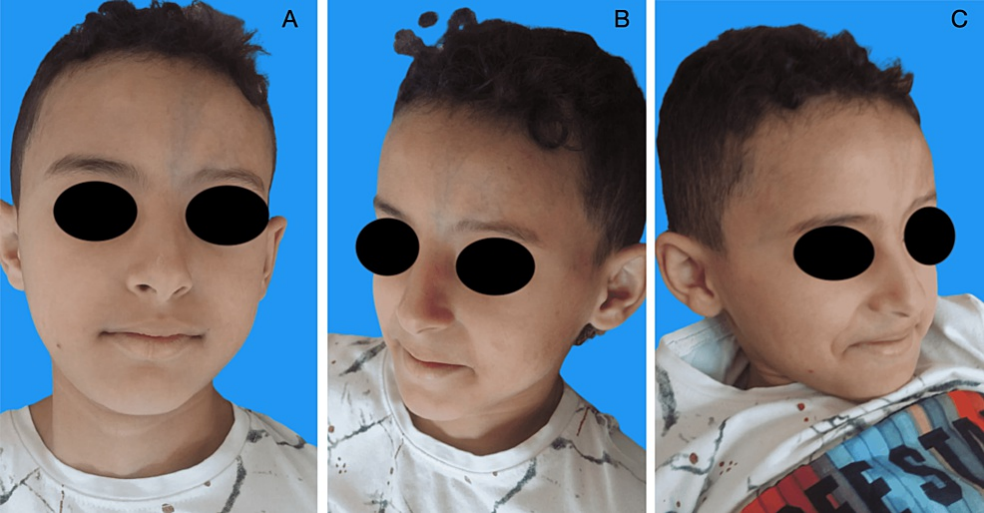
Postoperative front (A), 3/4 left (B), and right (C) photographs (seven months later) of patient 1

**Figure 9:**
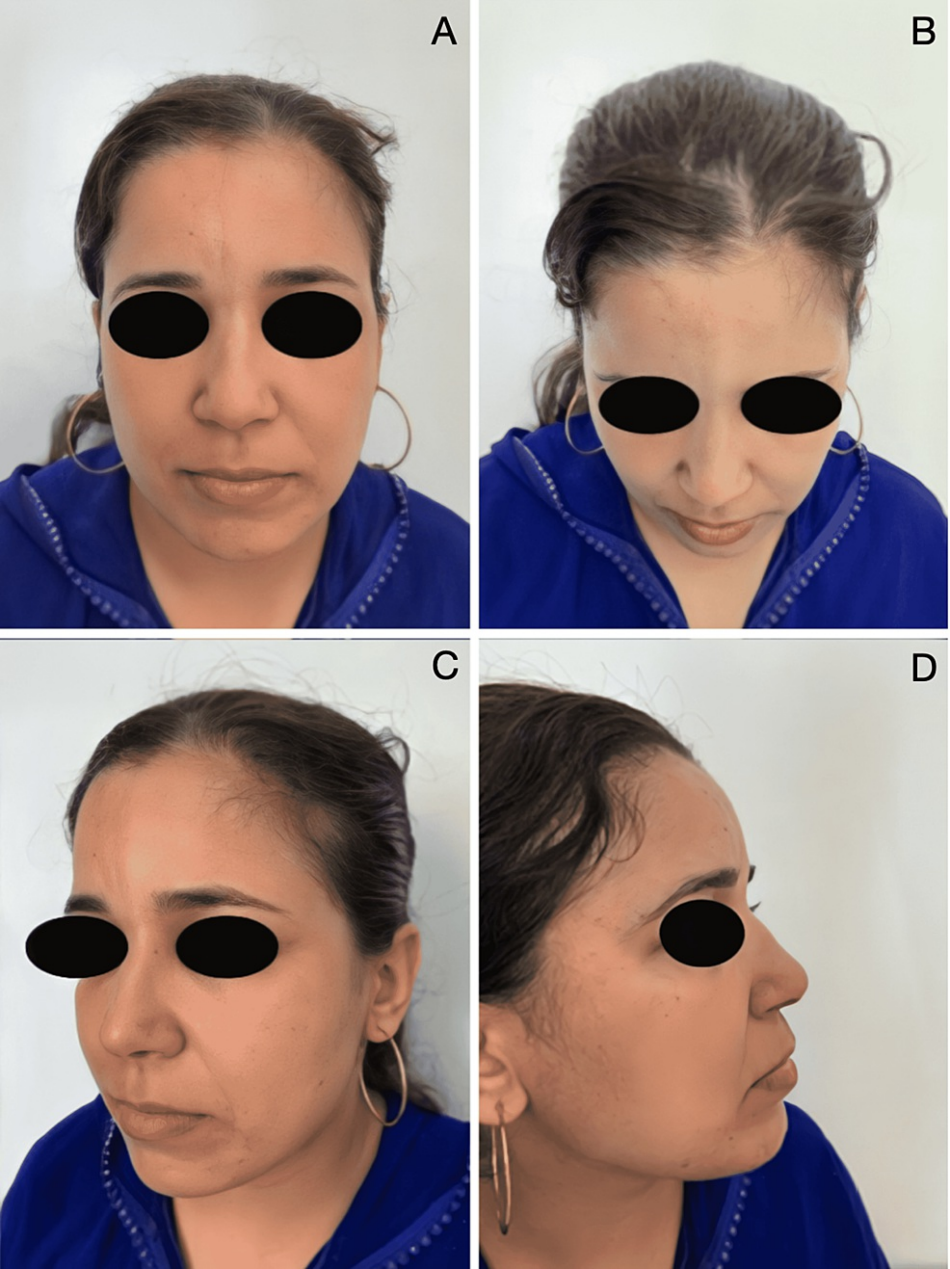
Late postoperative front (A), plunging view (B), 3/4 left (C), and profile right (D) photographs (nine years later) of patient 2

For both patients, the postoperative course was simple and no complications were noted (no necrosis, infection, hematoma, or hemorrhage). The aesthetic aspect was very satisfactory and stable, with a decline of seven months for the child (Figure 8), and nine years for the young woman (Figure 9).

The jury that answered the questionnaire was composed of a panel of 21 specialists, including nine dermatologists, six internists, and six plastic surgeons (Tables 1, 2).

**Table 1:** Preoperative and postoperative averages of patient 1

Patient 1	Preoperative average	Postoperative average
Volume	4.57/10	7.80/10
Skin quality	5.66/10	7.57/10
Symmetry	3.61/10	7.14/10

**Table 2:** Preoperative and postoperative averages of patient 2

Patient 2	Preoperative average	Postoperative average
Volume	3.8/10	7.66/10
Skin quality	3.38/10	7.9/10
Symmetry	4.80/10	7.14/10

The panel’s average score for overall aesthetic improvement was 8.33/10 for our first patient, and 8.76/10 for the second patient.

The patients described the psychological impact of this condition on their lives as significant for one and utterly stigmatizing for the other. Texture, self-perception, and the perception of others were rated “very good.” Skin color was rated “good” by both patients. Sensitivity in the corrected area was rated “good” by the young woman and “very good” by the child.

No sequelae in the donor area were reported by either patient. The young woman reported that her color, texture, and volume were stable since the intervention, while the child’s results, according to his mother, decreased over time. The time of stabilization of the graft for the child was three months, while the young woman reported no change in the grafted area since the intervention.

The overall aesthetic improvement was rated 8/10 by the child’s mother and 9/10 by the young woman. Both patients confirmed the improvement in their psychological comfort and social life as a result of the intervention and rated it 10/10.

The Lo-SCAT scoring grid allowed us to compare the scores in the preoperative and postoperative periods (Tables 3, 4).

**Table 3:** Lo-SCAT standard table for scoring scleroderma’s cutaneous activity Lo-SCAT: Localized Scleroderma Cutaneous Assessment Tool; Lo-SAI: Localized Scleroderma Skin Activity Index.

Lo-SCAT	Lo-SAI		
	New/enlarged (past month)	Erythema	Induration (skin swelling at edge)
	0 = none	0 = none	0 = none
	3 = N/E	1 = pink	1 = mild
		2 = red	2 = moderate
		3 = dark red/violaceous	3 = marked

**Table 4:** Lo-SCAT standard table for scoring scleroderma’s cutaneous damage Lo-SCAT: Localized Scleroderma Cutaneous Assessment Tool; Lo-SDI: Localized Scleroderma Skin Damage Index.

Lo-SCAT	Lo-SDI			
	Dermal atrophy	Sub Q/deep atrophy	Dyspigmentation (hyper or hypo)	Skin thickness (at center)
	0 = none	0 = none	0 = none	0 = none
	1 = shiny	1 = flat	1 = mild	1 = mild
	2 = visible vessels	2 = concave	2 = moderate	2 = moderate
	3 = cliff drop	3 = marked	3 = marked	3 = marked

The Lo-SCAT score measured a two-point drop out of 9 in activity, where the Localized Scleroderma Skin Activity (Lo-SAI) went from 3/9 preoperatively to 1/9 postoperatively for both patients. There was a four-point decrease out of 12 in pathology-related damage for the first patient, of which Localized Scleroderma Skin Damage Index (Lo-SDI) decreased from 7/12 preoperatively to 3/12 postoperatively. A five-point decrease out of 12 was recorded for the second patient, where the Lo-SDI dropped from 9 preoperatively to 3 postoperatively.

## Discussion

The diagnosis of morphea en coup de sabre is most often made at an advanced pathological stage. This diagnostic delay occurs because the clinical manifestations from the onset are polymorphic and not specific. Because of its occurrence in young populations and its very visible location, en coup de sabre is a socially stigmatizing pathological condition that justifies aesthetic correction. This was confirmed by our patients, who both described the psychological impact of this lesion on their lives as significant for the child and utterly stigmatizing for the young woman.

To date, various methods of dealing with sequelae induced by this pathology have been reported; nevertheless, there are some constraints and limitations specific to each one. First, the excision technique consists of ablation of the lesion tissue, followed by suturing the healthy edges together. This procedure has the disadvantage of inducing new scar formation and all the hazards of healing in an inflammatory area [4,5]. Bone grafting has also been used by some authors in scleroderma filling, resulting in the loss of bone substance and morbidity of the donor site [4,5]. Nevertheless, we believe that using bone alone does not provide the flexibility and texture of subcutaneous tissue, even though it provides a filling. Filling with BoneSource using hydroxyapatite cement could be an alternative for filling major bone defects and sparing the harvesting of bone from another site. Although easy and biocompatible, it still has the disadvantage of causing infection and being a less flexible and natural reconstruction [6].

Some authors have also suggested the injection of hyaluronic acid for filling. This material is especially effective for superficial skin atrophy without affecting the underlying structures [7]. However, its main limitations are its high resorption rate, its need for repeated injections, and its high cost [5]. The Medpor porous polyethylene implant has also been used to correct midface lipoatrophy lesions in HIV patients [8], and linear scleroderma en coup de sabre in others. When associated with an autologous fat graft, it provides satisfactory results in both situations in terms of aesthetic filling [9]. However, the potential for an immune reaction, the risk of infection, and its high cost are limitations to be considered in this procedure [4,5].

Finally, composite dermal-hypodermal grafting, which is increasingly being given up, has many drawbacks. Apart from the adequacy of the graft defect size, which is difficult to predict, this technique also has the disadvantage of the risk of revascularization and its subsequent absorption [5]. Autologous adipocyte transfer, as used in this study, is a simple technique applicable at no extra cost, with few sequelae at the donor site. The procedure leaves minimal scars on the recipient and donor sites and can be performed under local anesthesia within a short operating time, and with a rapid recovery rate [4].

This was confirmed in our study by the total absence of sequelae, such as pain, deformity, dysesthesia, dyschromia, or asymmetry in the different harvesting areas, and uncomplicated postoperative follow-up in both patients. Indeed, in recent years, autologous adipocyte transplants have shown particularly reliable results in improving skin suppleness and motricity in spots destroyed by atrophy and fibrosis in patients with scleroderma, particularly on the face [10].

Our study allowed us to validate the restructuring effect of adipose tissue through objective evaluation by a panel of specialists. There were improvements in the following parameters: volume was estimated at 33% for patient 1 and 33.5% for patient 2. Skin quality was estimated at 20% for patient 1 and 44.5% for patient 2. Skin color was estimated at 40% for patient 1 and 22.5% for patient 2. The percentage attributed by the specialists to the overall aesthetic improvement was 84% for the first patient and 88.5% for the second. This shows the undeniable qualities of adipose tissue for the morphological correction of lesions caused by morphea en coup de sabre.

Adipose tissue cannot be restricted to the role of a biological filling material since it has regenerative virtues attributable to the ability of its mesenchymal stem cells to secrete angiogenic and immunomodulator factors that activate tissue regeneration. Indeed, our patients had a decline in the activity and damage caused by their pathology because of adipocyte transplantation. The subcutaneous depression was not only filled but also improved in terms of the trophic qualities of the skin. This positive evolution is evidenced by the Lo-SCAT score measured in each patient pre- and postoperatively, with a two-point decrease out of 9 in the activity for both patients and a four-point decrease out of 12 in damage caused by the pathology for the first patient and a five-point decrease out of 12 for the second. Therefore, fat cell transplantation not only represents a palliative treatment for en coup de sabre but also a potentially curative treatment. Our results are very encouraging, even though our study is limited to two clinical cases.

Numerous studies have already highlighted the immunomodulatory role of autologous adipose tissue in vitro. In 2001, researchers from the Laboratory of Regenerative and Restorative Bioengineering at the University of Los Angeles and researchers from the Department of Plastic and Reconstructive Surgery at the University of Pittsburgh took up the challenge of demonstrating that it was possible to isolate a population of mesenchymal stem cells from human adipose tissue. This experiment aimed to highlight the regenerative potential of adipose tissue, which is far superior to that of less accessible tissues in terms of quantity, such as bone marrow, or even the ethically controversial case of embryonic stem cells [11,12]. Other experiments have highlighted the proliferative and differentiation potential of mesenchymal stem cells in adipocytes, chondrocytes, and osteoblastic lineages, opening up a wide range of therapeutic indications, particularly in regenerative and reparative surgery [11,13].

The treatment of a destructive pathology of the subcutaneous tissue, such as morphea en coup de sabre, requires mature adipocytes and, preferably, stem cells with adipocyte differentiation potential to obtain an optimal and durable result [11]. After transplantation, the graft is destined for resorption of 20-90% of its initial volume [10,14]. Two theories concerning the fate of adipose tissue have been proposed. The “graft survival theory,” described by Peer and advocated by Coleman and many other authors, states that the graft survives by imbibition until neovascularization from the recipient site occurs [14,15]. In contrast, the “graft replacement theory” proposed by Neuhof and Hirshfeld in 1923 [16] has gained importance; it holds that very few adipocytes survive the grafting process. The suffering of fat cells leads to an influx of immune cells, and they are replaced by newly differentiated autologous stem cells co-transplanted with the graft [14]. In our study, we were not able to perform a histological study to observe the evolution of injected fat tissue. However, from the follow-up of patient 2, the graft was stabilized and did not resorb over the nine years of follow-up, while for patient 1, the resorption was estimated at 30% after seven months.

Based on the assumption that adipose tissue resorption is the result of ischemia and lack of neoangiogenesis, several studies have been conducted to determine ways of increasing graft viability through the addition of anabolic agents, stromal vascular fraction, and vasodilators. Despite this, many clinicians do not advocate the addition of biological activators to harvested adipose tissue. Among them is Coleman, who opposed the addition of chemicals, hormones, drugs, or any other foreign substance to the tissue. He assumed that a good result was based on the correct application of his techniques under the right conditions while stressing the importance of preventing chemical or mechanical damage that could be inflicted on sensitive fat tissue [17]. In both patients, we used the adipocyte transplant technique without any additions, and the morphological result after a single session was adjudged satisfactory by both the panel of doctors and the patients themselves. Moreover, the young woman enjoyed nine years of steady results after the intervention.

This raises another question concerning the fate of graft in growing children. Adipocyte transfer is presumed the best treatment since it has numerous advantages in terms of ease of access, practicality, and cost [15]. However, when it is performed at an early age, it is natural to ask questions about the fate of the graft within a growing body. This is more so when, in our study, the patient with the least satisfactory results in terms of graft sustainability was a child. In a prospective study carried out on a group of 12 children who had received autologous adipose tissue grafts, the retention of the graft after one year was similar to that of adults, despite local and general inter-individual variations, according to a precise three-dimensional evaluation system [18]. This would theoretically refute the hypothesis that growth has an impact on graft survival. However, longer-term studies should be performed to determine the effects of adolescence, weight variations, and other environmental factors on graft evolution in children throughout their growth. This review of the literature affirms the extraordinary regenerative potential of adipose tissue because of the presence of mesenchymal stem cell progenitors. This discovery constitutes new hope for potentially curative treatment for this disease, with extremely limited therapeutic perspectives. However, the first problem arises, namely, the delay in diagnosis concerning this condition. Very often unrecognized by patients and almost always diagnosed at a sequential stage, the handling of morphea en coup de sabre is most often a matter of aesthetic correction. Even when the suspicion of en coup de sabre is raised at the beginning of the evolution of the pathology, skin biopsy, a diagnostic confirmation tool, is characteristic but not pathognomonic of this pathology at an early stage.

This raises the second issue of which attitude to adopt in this case. Based on clinical features, such as the location and morphology of the lesion and histological features associated with the presence of anomalies that are characteristic of localized scleroderma, is it possible to affirm the diagnosis of morphea and thereby be able to undertake the necessary measures? Specific clinical trials for morphea should be conducted to answer the question of the contribution of stem cells of adipose origin during the active phase. Could the immunomodulatory abilities of adipose tissue prevent the initiation of the pathological cascade?

## Conclusions

Morphea en coup de sabre is a rare pathology that causes significant aesthetic damage to the face and for which, to date, no curative treatment has been found yet. However, through our study, autologous transfer of adipose tissue appears to be a promising therapeutic approach for both morphological and functional improvements to this pathology. Therefore, future research should focus on refining current techniques and answering the questions we have raised aimed at improving the treatment of this disease.
